# Demographic, health, and prognostic characteristics of Australians with liver cancer: a cohort study of linked data in New South Wales for informing cancer control

**DOI:** 10.1186/s12889-023-16809-y

**Published:** 2023-10-09

**Authors:** David Roder, David Banham, Jacob George, Shelley Rushton, Tracey O’Brien

**Affiliations:** 1 Cancer Institute NSW, NSW Government, Sydney, NSW Australia; 2 Storr Liver Centre, The Westmead Institute of Medical research, Westmead Hospital and University of Sydney, Sydney, NSW Australia

**Keywords:** Liver cancer, Risk factors – demographic factors, Data linkage

## Abstract

**Background:**

Australian age-standardized incidence and death rates for liver cancer are lower than world averages, but increasing as in other economically advanced western countries. World Health Organization emphasizes the need to address sociodemographic disparities in cancer risk. A more detailed sociodemographic risk profiling was undertaken for liver cancer in New South Wales (NSW) by diagnostic stage, than possible with NSW Cancer Registry (NSWCR) alone, by incorporating linked data from the Australian Bureau of Statistics (ABS). The purpose was to inform targeting and monitoring of cancer services.

**Methods:**

The ABS manages the Multi-Agency Data Integration Project (MADIP) which includes a wide range of health, educational, welfare, census, and employment data. These data were linked at person level to NSWCR liver cancer registrations for the period post 2016 census to December 2018. De-identified data were analyzed. Sex-specific age-adjusted odds ratios (95%CIs) of liver cancer were derived using logistic regression by age, country of birth, residential remoteness, proficiency in spoken English, household income, employment status, occupation type, educational attainment, sole person household, joblessness, socioeconomic status, disability status, multimorbidity, and other health-related factors, including GP consultations. These data complement the less detailed sociodemographic data available from the NSWCR, with alignment of numerators and population denominators for accurate risk assessment.

**Results:**

Results indicate liver cancer disproportionately affects population members already experiencing excess social and health disadvantage. Examples where 95% confidence intervals of odds ratios of liver cancer were elevated included having poor English-speaking proficiency, limited education, housing authority tenancy, living in sole-person households, having disabilities, multiple medicated conditions, and being carers of people with a disability. Also, odds of liver cancer were higher in more remote regions outside major cities, and in males, with higher odds of more advanced cancer stages (degrees of spread) at diagnosis in more remote regions.

**Conclusions:**

Linked data enabled more detailed risk profiling than previously possible. This will support the targeting of cancer services and benchmarking.

**Supplementary Information:**

The online version contains supplementary material available at 10.1186/s12889-023-16809-y.

## Background

Incidence and mortality from liver and intra-hepatic bile duct cancer (ICD-10-C22) are lower in Australia than the world average by 34% and 52% respectively, and much lower than in Africa and Asia [1]. Nonetheless, as in other economically advanced western countries, increases have occurred in recent decades [1–3]. Between 1982 and 1989, when national incidence data were first available, and 2015–2019, the age-standardized Australia-wide incidence increased by about 313%, with a corresponding mortality increase of 169%.^3^ In 2021, an estimated 2,832 Australians (72% males) were diagnosed with liver cancer and 2,424 (66% males) died from this cancer [4.

World Health Organization emphasizes the need to address sociodemographic disparities in cancer incidence, as reported in Australia for liver cancer [3–5]. This is reflected in national and international declarations and strategies, [6–8] and supported by the Australia Public Health Association, Australian and NSW Governments, and the NSW Cancer Plan [9–11].

Viral hepatitis B and C infections are major causes of liver cancer and contribute to upward trends in incidence trends [5]. Research in Australia indicates that almost half these cancers are associated with hepatitis B or C infection [5]. Other risk factors include type 2 diabetes mellitus, overweight and obesity, high alcohol consumption, tobacco smoking, illicit drug use, unprotected sex, medical conditions such as metabolic dysfunction associated fatty liver disease, and hereditary haemochromatosis [5, 6]. In some low-income countries, humidity and suboptimal storage of foodstuffs may also contribute to risk through increased aflatoxin contamination of food products [6].

Like most cancers, incidence of liver cancer increases and survival decreases in Australia with age [3]. Males are more frequently affected, with elevated male to female sex ratios of around 3.3 to one [3]. The age-sex standardized rate varies by socioeconomic status at about 52% higher in the most than least disadvantaged socioeconomic quintile of residential areas [7]. Compared with the non-Indigenous population, the age-standardized incidence rate has been reported at 132% higher for Aboriginal and Torres Strait Islander people [7]. Residents of major cities and remote/very remote country areas are reported to be at a 20–25% higher risk than those living in regional country areas [7].

The study aim is to examine sociodemographic disparities in risk of liver cancer, and of more advanced stage at diagnosis, in greater detail to inform the planning and benchmarking of NSW cancer services. This has become possible through linking NSW Cancer Registry (NSWCR) data with Australian Bureau of Statistics (ABS) data extracts for the post-2016 census diagnostic period.

## Methods

### Study design

A retrospective cohort design covered all NSW residents included in the 2016 census when aged 18 + years. The study period was from September 2016 to December 2018. The study was designed to gain more detailed evidence of sociodemographic disparities in risk of liver cancer, and of more advanced diagnostic stage, to inform the planning and benchmarking of NSW cancer services. In particular the purpose was to indicate groups at elevated risk of liver cancer who may require additional attention in service planning and delivery. This was not a causal study.

### Data sources

NSWCR liver cancer registrations were linked with sociodemographic and health data obtained through the Australian Bureau of Statistics (ABS) Multi-Agency Data Integration Project (MADIP), [12] for the study period. NSWCR provided dates of liver cancer diagnoses and stages (degree of spread) at diagnosis. Sociodemographic data were obtained through MADIP from the 2016 Australian census and other administrative sources, including data on health and educational status, ethnicity, household income, and employment [12]. Universal health insurance claims data from the Medicare Benefits Schedule (MBS) and Pharmaceutical Benefits Scheme (PBS) were also obtained [12].

### Data management

MADIP included a unique Person Linkage Spine (PLS) for any person recorded in the Australian Medicare Consumer Directory, Centrelink or Taxation datasets between 2006 and 2016, which enabled the ABS, as the accredited Integrating Authority, to link multiple datasets. The present study included records for all adults aged 18 years or more in NSW at time of the Census (August 2016) and recorded on the PLS. Exclusions comprised those without a PLS, and those with a first invasive cancer diagnosis other than cancer of the liver and intrahepatic bile ducts (ICD-10-AM C22) occurring between September-2016 and December-2018 [13].

### Data variables

The NSWCR provided data on degree of cancer spread (local, regional, and distant/unknown). Census records provided socio-demographic data on age, sex, geographic residential remoteness using the Accessibility and Remoteness Index of Australia (ARIA), Aboriginal self-identification, country of birth, and household composition [12]. Census data further indicated socio-economic position as indexed by the ABS Socio-economic Indexes for Areas (SEIFA) and specifically, the Index of Relative Socio-Economic Disadvantage (IRSD) [14, 15].

PBS records available through MADIP were used for each person in the 12-months before liver cancer diagnosis, or the 12-months before the census enumeration for those without a cancer diagnosis. PBS extracts included the Anatomical Therapeutic Classification (ATC) of prescribed medications enabling categorization of medicated conditions using the Rx Risk comorbidity index [16]. To assess the potential for selection bias within the entire enumerated population, we examined census variables for systematic variations according to whether recorded on the PLS.

The linked NSW data with identifiers removed were stored in a high-security ABS repository (DATALAB) for analysis by remote access [12]. The primary outcome variable was first diagnosis of liver cancer following the census, from September 2016 to December 2018 (= 1), as opposed to no first cancer diagnosis (= 0). The secondary outcome variable was the degree of spread among those diagnosed with liver cancer, classified as distant/unknown disease (= 1) compared with localized/regional extent of disease (= 0). These binary classifications were used to gain sufficient numbers to avoid prohibitively small cell counts, and increase interpretability of results. Unknown degree of spread was combined with distant spread because its disease-specific survival was previously shown to be lower than for localized/regional spread (i.e., its survival was more akin to survival for distant spread) [17].

Socio-demographic variables included age at census, arranged in categories for tabling results and as a continuous measure in multivariable models, sex, geographic remoteness (major city, inner regional, outer regional/remote), ancestry (based on country of birth grouped as Australia; China; Greece; Italy; Lebanon; New Zealand; the Philippines; the United Kingdom; Vietnam; “other mainly English speaking” and “other mainly non-English speaking” countries), and lone occupant household. Socio-economic disadvantage covariates included area-level IRSD quintiles based on Statistical Areas (SA2).

We included each of the discrete variables underpinning the IRSD as dichotomized variables following ABS methods [17]. Those variables included poor English language proficiency, low household income, core function-limiting disability, employment status and occupation (drivers and laborers), education attainment, a household with children, resident household numbers, and rental through a housing authority. Additional data on housing, such as overcrowding, household internet connection, and car ownership, were not available through MADIP.

### Data quality

Registry data achieve high quality data standards as recommended for liver cancer by the International Agency for Research on Cancer (Volume 11), Cancer Incidence in Five Continents for 2008–2012 diagnoses) [13]. A key index of data accuracy is the percentage of cancers microscopically verified (MV%) which is high for Australia by world standards [13]. In Australia, NSW reported a higher MV% for liver cancer than other States and Territories for males and females collectively, and higher than for Australia overall both for males and females [13].

### Statistical analysis

Analyses for each outcome were undertaken for males and females separately in two steps. In step 1, cross-tabulations, and odds ratios with 95% confidence intervals derived from multiple logistic regressions, were used to compare characteristics of cohort members according to whether they were subsequently diagnosed with liver cancer. Also, within cohort members diagnosed with liver cancer, similar comparisons were made by degree of spread (i.e., distant/unknown degree of spread compared with localized/regional) [18, 19]. This was undertaken for each socio-demographic and health status variable. Odds ratios were age-adjusted, as initial examination showed age was strongly associated with each variable (e.g., 93% of the linked liver cancer cohort compared with 44% of the unlinked were aged 50 + years; and proportions of liver cancers with distant/unknown degree of spread increased from 35.2% for ages < 50 years to 50.6% for ages 80 + years for males and females in aggregate) (Tables 1 and 4).

**Table 1:** Age distribution of NSW liver cancer cases (Sept 2016-Dec 2018) and NSW community controls (2016 census) *

Age at diagnosis (yrs.)	Number of liver cancer cases on NSWCR (%)	Number of community controls (%)	Mann-Whitney U test P value
Sex (males)			
18–39	17 (2.0)	975 561 (39.3)	MWp < 0.001
40–44	15 (1.7)	219 788 (8.9)	
45–49	33 (3.8)	212 552 (8.6)	
50–54	96 (11.0)	211 395 (8.5)	
55–59	133 (15.3)	201 199 (8.1)	
60–64	176 (20.2)	178 151 (7.2)	
65–69	140 (16.1)	163 378 (6.6)	
70–74	104 (12.0)	122 328 (4.9)	
75–79	70 (8.0)	87 180 (3.5)	
80–84	50 (5.7)	58 262 (2.3)	
85+	36 (4.1)	51 075 (2.1)	
Total	870 (100)	2 480 869 (100)	
Sex (females)			
18–44	13 (4.1)	1 371 183 (46.6)	MWp < 0.001
45–49	10 (3.2)	261 286 (8.9)	
50–54	16 (5.1)	247 658 (8.4)	
55–59	26 (8.3)	237 632 (8.1)	
60–64	41 (13.0)	210 591 (7.2)	
65–69	61 (19.4)	190 470 (6.5)	
70–74	31 (9.8)	143 805 (4.9)	
75–79	37 (11.7)	107 481 (3.7)	
80–84	42 (13.3)	78 982 (2.7)	
85+	38 (12.1)	92 176 (3.1)	
Total	315 (100)	2 941 264 (100)	

*Cancer cases–NSW Cancer Registry; Community controls–ABS census

**Table 2:** Age-adjusted odds ratios (95% CI) of males having a liver cancer diagnosed during Sept 2016 – Dec 2018, according to sociodemographic predictors and medicated conditions: linked NSW Cancer Registry and MADIP data

Characteristic	Numbers in liver cancer cohort (Col %)	Number of community controls (Col %)	Age-adjusted odds ratios (95% CI)	Multivariate-adjusted odds ratios (95% CI)
**Total**	870 (100)	2 480 869 (100)	-	-
**Residential remoteness**:				
Major cities	633 (72.8)	1 864 809 (75.2)	1.00	
Inner regional	176 (20.2)	463 063 (18.7)	0.88 (0.75, 1.05)	
Outer regional/remote	61 (7.0)	152 997 (6.2)	0.90 (0.69, 1.17)	
**Country of birth**:				
Australia	486 (55.9)	1 579 161 (63.7)	1.00	1.00
China	41 (4.7)	84 343 (3.4)	1.87 (1.36, 2.57)	2.25 (1.56, 3.24)
Italy, Greece, Lebanon	41 (4.7)	62 226 (2.5)	1.16 (0.84, 1.59)	0.92 (0.66, 1.29)
NZ, Philippines, Vietnam	55 (6.3)	109 812 (4.4)	1.86 (1.41, 2.46)	1.80 (1.35, 2.41)
United Kingdom	60 (6.9)	122 907 (5.0)	1.09 (0.83, 1.42)	1.25 (0.95, 1.63)
Other English speaking	25 (2.9)	78 593 (3.2)	0.85 (0.57,1.28)	1.05 (0.70, 1.57)
Other Non-English speaking	162 (18.6)	443 827 (17.9)	1.25 (1.05, 1.50)	1.20 (0.99, 1.45)
**English speaking proficiency**:				
Poor	97 (11.1)	96 270 (3.9)	2.11 (1.70, 2.61)	1.41 (1.09, 1.83)
Not poor	773 (88.9)	2 384 599 (96.1)	1.00	1.00
**Low-income household (<$26,000)**:				
Yes	277 (31.8)	380 322 (15.3)	1.64 (1.42, 1.90)	1.20 (1.03, 1.41)
No	593 (68.2)	2 100 547 (84.7)	1.00	1.00
**Unemployed**:				
Yes	24 (2.8)	103 361 (4.2)	1.29 (0.86, 1.95)	
No	846 (97.2)	2 377 508 (95.8)	1.00	
**Educational level (< year 12)**:				
Yes	330 (37.9)	503 305 (20.3)	1.66 (1.44, 1.90)	1.25 (1.08, 1.44)
No	540 (62.1)	1 977 564 (79.7)	1.00	1.00
**No education**:				
Yes	24 (2.8)	19 676 (0.8)	2.03 (1.35, 3.05)	
No	846 (97.2)	2 461 193 (99.2)	1.00	
**Labourer (occupation)**:				
Yes	53 (6.1)	173 903 (7.0)	1.51 (1.14, 2.01)	1.78 (1.33, 2.37)
No	817 (93.9)	2 306 966 (93.0)	1.00	1.00
**Driver (occupation)**:				
Yes	61 (7.0)	176 149 (7.1)	1.47 (1.13, 1.91)	1.72 (1.30, 2.56)
No	809 (93.0)	2 304 720 (92.9)	1.00	1.00
**Renting from housing authority**:				
Yes	80 (9.2)	68 446 (2.8)	3.22 (2.55, 4.05)	1.72 (1.35, 2.21)
No	790 (90.8)	2 412 423 (97.2)	1.00	1.00
**Sole person household**:				
**Yes**	181 (20.8)	267 997 (10.8)	1.50 (1.27, 1.77)	1.43 (1.20, 1.70)
**No**	689 (79.2)	2 212 872 (89.2)	1.00	1.00
**Jobless household (with children)**:				
Yes	18 (2.1)	48 080 (1.9)	1.73 (1.08, 2.77)	
No	852 (97.9)	2 432 789 (98.1)	1.00	
**Disability (6 + months) (age < 70 yrs.)**:				
Yes	95 (10.9)	78 832 (3.2)	3.99 (3.22, 4.95)	2.05 (1.67, 2.64)
No	775 (89.1)	2 402 937 (96.8)	1.00	1.00
**Carer of person with disability**:				
Yes	86 (9.9)	258 221 (10.4)	0.83 (0.66, 1.03)	
No	784 (90.1)	2 222 648 (89.6)	1.00	
**SA2 area disadvantage**:				
Most disadvantage (Q1)	248 (28.5)	538 029 (21.7)	1.77 (1.44, 2.18)	
Quintile (Q2)	205 (23.6)	520 226 (21.0)	1.47 (1.18, 1.83)	
Quintile (Q3)	174 (20.0)	494 530 (19.9)	1.46 (1.17, 1.83)	
Quintile (Q4)	107 (12.3)	363 968 (14.7)	1.25 (0.97, 1.61)	
Least disadvantage (Q5)	136 (15.6)	564 116 (22.7)	1.00	
**No. of conditions medicated**:				
0	67 (7.7)	1 177 607 (47.5)	0.17 (0.13, 0.24)	0.16 (0.12, 0.22)
1	77 (8.9)	448 023 (18.1)	0.44 (0.33, 0.59)	0.39 (0.29, 0.53)
2	126 (14.5)	280 922 (11.3)	1.00	0.84 (0.65, 1.08)
3	118 (13.6)	190 325 (7.7)	1.22 (0.95, 1.57)	1.00
4	124 (14.3)	133 434 (5.4)	1.67 (1.29, 2.14)	1.34 (1.04, 1.72)
5	104 (12.0)	93 082 (3.8)	1.87 (1.44, 2.45)	1.48 (1.13, 1.93)
6–7	156 (17.9)	103 665 (4.2)	2.37 (1.85, 3.03)	1.84 (1.44, 2.35)
8+	98 (11.3)	53 811 (2.2)	2.72 (2.06, 3.59)	2.02 (1.54, 2.67)

In step 2, multivariable analyses were undertaken, starting with all covariates, then purposefully removing the least-contributing covariates (using Wald statistic p-values of > 0.2 as a guide), and refitting the model with remaining covariates until deriving a main effects model where each retained covariate substantially contributed [18, 19]. Potential for co-linearity among predictor variables was tested using variance inflation factors to ensure it was within accepted limits. Data preparation and analyses were undertaken using Stata 17 within the ABS DATALAB facility.

Step 1 and 2 analyses were undertaken for all variables where data were collected prior to the liver cancer diagnoses (Tables 2-4). Preliminary age-adjusted analyses were also undertaken for supplementary variables shown in Appendix A.

**Table 3:** Age-adjusted odds ratios (95% CI) of females having a liver cancer diagnosed during Sept 2016 – Dec 2018, according to sociodemographic predictors and medicated conditions: linked NSW Cancer Registry and MADIP data

Characteristic	Numbers in liver cancer cohort (%)	Number of community controls (%)	Age-adjusted odds ratios (95% CI)	Multivariate-adjusted odds ratios (95% CI)
**Total**	315 (100)	2 941 264 (100)	----	----
**Residential remoteness**:				
Major cities	238 (75.6)	2 178 998 (74.1)	1.00	
Inner regional	63 (20.0)	565 118 (19.2)	0.81 (0.61, 1.06)	
Outer regional/remote	14 (4.4)	197 148 (6.7)	0.54 (0.32, 0.83)	
**Country of birth**:				
Australia	162 (51.4)	1 898 588 (64.6)	1.00	1.00
China	15 (4.8)	112 424 (3.8)	2.22 (1.31, 3.77)	1.88 (1.01, 3.48)
Italy, Greece, Lebanon	16 (5.1)	62 017 (2.1)	1.59 (0.95, 2.66)	1.04 (0.60, 1.81)
NZ, Philippines, Vietnam	21 (6.7)	147 565 (5.0)	2.19 (1.38, 3.45)	1.88 (1.16, 3.05)
United Kingdom	24 (7.6)	132 267 (4.5)	1.41 (0.92, 2.17)	1.52 (0.99, 2.34)
Other English speaking	13 (4.1)	91 408 (3.1)	1.45 (0.82, 2.54)	1.67 (0.94, 2.94)
Other Non-English speaking	64 (20.3)	496 995 (16.9)	1.59 (1.19, 2.13)	1.36 (0.98, 1.88)
**English speaking proficiency**:				
Poor	53 (16.8)	141 038 (4.8)	2.58 (1.92, 1.34)	1.78 (1.20, 2.63)
Not poor	262 (83.2)	2 800 226 (95.2)	1.00	1.00
**Low-income household (<$26,000)**:				
Yes	94 (29.8)	524 829 (17.8)	1.22 (0.95, 1.56)	
No	221 (70.2)	2 416 435 (82.2)	1.00	
**Educational level (< year 12)**:				
Yes	157 (49.8)	725 596 (24.7)	1.42 (1.13, 1.79)	
No	158 (50.2)	2 215 668 (75.3)	1.00	
**No education**:				
Yes	18 (5.7)	28 394 (1.0)	2.84 (1.76, 4.59)	
No	297 (94.3)	2 912 870 (99.0)	1.00	
**Renting from housing authority**:				
Yes	28 (8.9)	94 843 (3.2)	2.56 (1.73, 3.77)	1.65 (1.10, 2.45)
No	287 (91.1)	2 846 421 (96.8)	1.00	1.00
**Sole person household**:				
**Yes**	80 (25.4)	368 677 (12.5)	1.03 (0.79, 1.34)	
**No**	235 (74.6)	2 572 587 (87.5)	1.00	
**Jobless household (with children)**:				
Yes	10 (3.2)	109 724 (3.7)	2.20 (1.16, 4.17)	
No	305 (96.8)	2 831 540 (96.3)	1.00	
**Sole parent household**				
Yes	12 (3.8)	175 648 (6.0)	1.57 (087, 2.83)	
No	303 (96.2)	2 765 616 (94.0)		
**Disability (6 + months/ages < 70 yrs.)**:				
Yes	23 (7.3)	87 634 (3.0)	3.17 (2.06, 4.88)	1.69 (1.08, 2.65)
No	292 (92.7)	2 853 630 (97.0)	1.00	1.00
**Carer of person with disability**:				
Yes	43 (13.7)	436 337 (14.8)	1.02 (0.73, 1.40)	
No	272 (86.3)	2 504 927 (85.2)	1.00	
**SA2 area disadvantage**:				
Most disadvantage (Q1)	101 (32.1)	614 637 (20.9)	1.66 (1.20, 2.28)	
Quintile (Q2)	66 (21.0)	615 418 (20.9)	1.05 (0.74, 1.49)	
Quintile (Q3)	51 (16.2)	581 338 (19.8)	0.98 (0.67, 1.43)	
Quintile (Q4)	38 (12.1)	454 648 (15.5)	0.97 (0.64, 1.46)	
Quintile (Q5)	59 (18.7)	675 223 (23.0)	1.00	
**No. of conditions medicated**:				
0	20 (6.3)	1 213 476 (41.3)	0.26 (0.15, 0.45)	0.17 (0.10, 0.30)
1	22 (7.0)	590 114 (20.1)	0.44 (0.26, 0.75)	0.30 (0.18, 0.49)
2	41 (13.0)	369 181 (12.6)	1.00	0.65 (0.43, 0.93)
3	55 (17.5)	246 909 (8.4)	1.58 (1.05, 2.37)	1.00
4	40 (12.7)	175 478 (6.0)	1.34 (0.86, 2.09)	0.83 (0.55, 1.26)
5	36 (11.4)	124 139 (4.2)	1.50 (0.95, 2.37)	0.92 (0.60, 1.40)
6–7	59 (18.7)	144 488 (4.9)	1.88 (1.24, 2.85)	1.14 (0.78, 1.66)
8+	42 (13.3)	77 479 (2.6)	2.29 (1.46, 3.58)	1.36 (0.90, 2.03)

**Table 4:** Number (%) of liver cancer cases by degree of spread (DOS) at diagnosis: NSW Cancer Registry, September 2016 to December 2018

Age at diagnosis (yrs.)	Number with localized/regional DOS (%)	Number with distant/unknown DOS (%)	Mann-Whitney U test P value
Sex (males)			
18–49	43 (66.2)	22 (33.8)	MWp = 0.004
50–59	151 (65.9)	78 (34.1)	
60–69	195 (61.7)	121 (38.3)	
70–79	95 (54.6)	79 (45.4)	
80+	45 (52.3)	41 (47.7)	
Total	529 (60.8)	341 (39.2)	
Sex (females)			
18–49	14 (60.9)	9 (39.1)	MWp = 0.038
50–59	26 (61.9)	16 (38.1)	
60–69	61 (59.8)	41 (40.2)	
70–79	36 (52.9)	32 (47.1)	
80+	37 (46.3)	43 (53.8)	
Total	174 (55.2)	141 (44.8)	

## Results

### Flow diagram for cohort selection

**Fig. 1:**
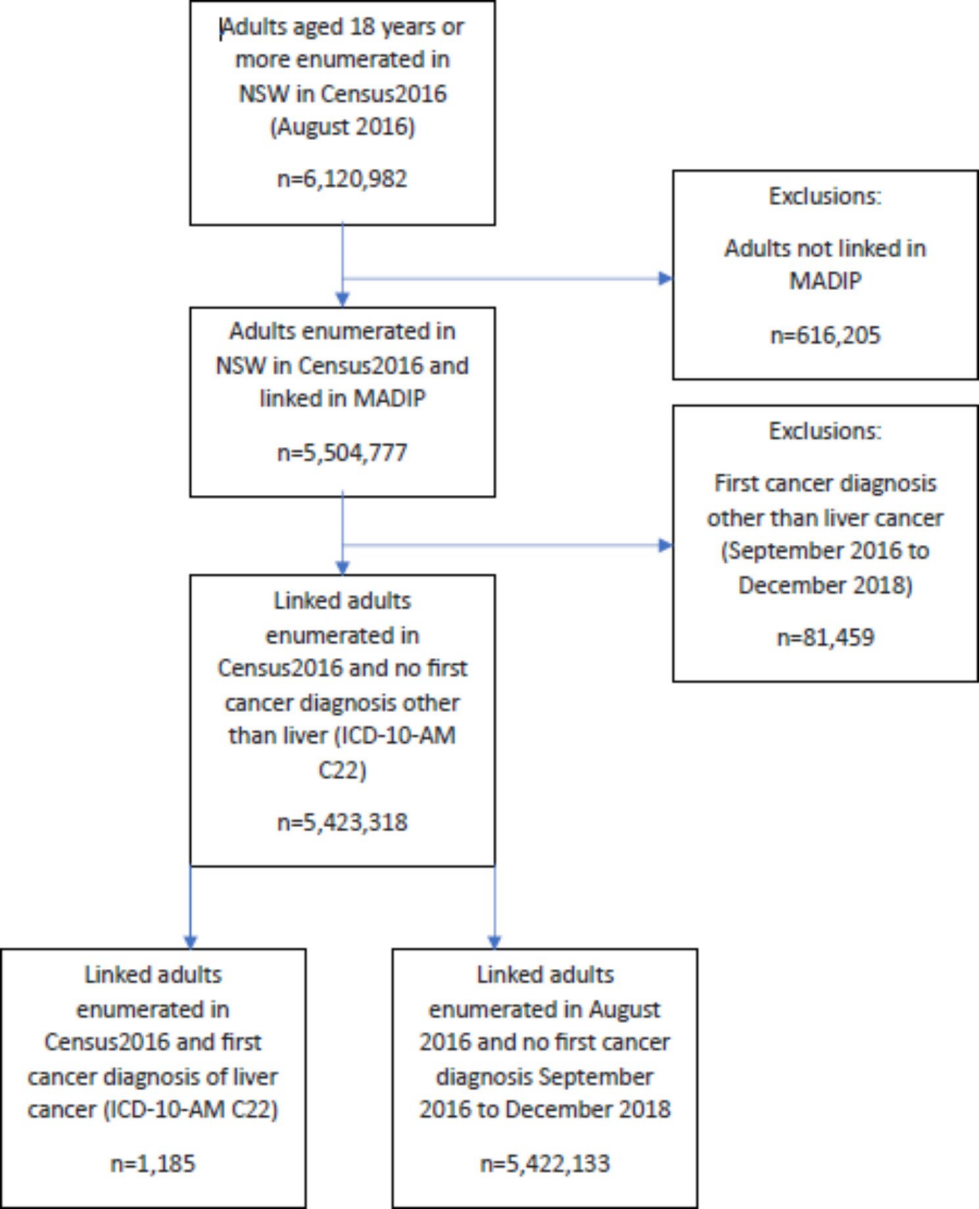
Cohort selection diagram

Census enumeration in NSW included 6,120,982 adults, 89.9% of whom were linked with a PLS link assigned (Fig. 1). Higher proportions of persons without a PLS link included males (11.1% of males versus 9.2% of females), the youngest (11.9%) and oldest adults (13.1%), those in remote areas (15.5%), and those from an unspecified, non-English speaking country of birth (29.1%). People first diagnosed with a cancer other than liver cancer were excluded. The resulting linked liver-cancer cohort (n = 1,185) was compared with other MADIP linked people in NSW for whom there was no evidence of liver cancer from the Cancer Registry (n = 5,422,133) (Fig. 1; Table 1).

### 1. Males - Adjusting only for age

Odds ratios for liver cancers in males are shown in Table 2  by: country of birth, with higher ratios for China at 1.87 (1.36, 2.57), New Zealand, Philippines and Vietnam in aggregate at 1.86 (1.41, 2.46), and “other non-English speaking” countries at 1.25 (1.05, 1.50), compared with Australian-born; and for residents with poor English-speaking proficiency, low-income households, educational achievement of less than year 12, those recording no education, and those with occupations recorded as labourer or driver. Elevated odds ratios were also seen for those renting from housing authorities, those living in a sole person household, or in a jobless household with children. Less socioeconomic disadvantage was associated with reduced odds of liver cancer. Other associations with elevated odds of liver cancer occurred when having a disability of at least 6-months duration when aged < 70 years, increased numbers of medicated conditions, receiving affective and antipsychotic medications, and with increased numbers of general practitioner consultations, evidence of health plans and mental health plans, and elevated numbers of chronic disease plans (Appendix A).

### Multivariable age-adjustment

Results indicated substantive elevated odds of liver cancer by country of birth for China at 2.25 (1.56, 3.24), and New Zealand, Philippines and Vietnam in aggregate at 1.80 (1.35, 2.41), when compared with Australian- born (Table 2). Elevated odds also applied for residents with poor English-speaking proficiency, low-income households, low educational attainment of under year 12, occupations of labourer or driver, renting from a housing authority, having a sole person household, having a disability of 6 + months when aged < 70 years, and having higher numbers of conditions medicated.

### 2. Females - Adjusting only for age

Odds ratios for liver cancer are shown in Table 3. A lower odds ratio at 0.54 (0.32, 0.83) was indicated for regional/remote residential areas than major cities. Other differences included elevated odds ratios by country of birth for China at 2.22 (1.31, 3.77); New Zealand, Philippines, and Vietnam collectively at 2.19 (1.38, 3.45); and other “mainly non-English speaking” countries at 1.59 (1.19, 2.13), when compared with Australian-born. Elevated age-adjusted odds ratios also applied for those with poor English-speaking proficiency, a low educational attainment below year 12, those recorded as having “no education”, those renting from a housing authority, those living in a jobless household with children, those having a disability of 6 + months duration when aged < 70 years, and the more socioeconomic disadvantaged, measured at individual household or residential SA2 level. Odds of liver cancer also increased with numbers of medicated conditions.

### Multivariable age-adjustment

These analyses indicated substantive elevated odds of liver cancer by country of birth for China at 1.88 (1.01, 4.48), and New Zealand, Philippines, and Vietnam collectively at 1.88 (1.16, 3.05), when compared with Australian-born (Table 3). Other elevations in odds ratios were indicated for poor English-speaking proficiency, renting from a housing authority, having a disability of 6 + months duration when aged < 70 years, and numbers of medicated conditions.

### Distribution of stage (degree of spread) by age

Localized/regional spread became less common and distant/unknown spread became more common with increasing age at diagnosis (p = 0.004 for males; p = 0.038 for females) (Table 4).

### Age-adjusted odds (95%CI) of distant/unknown versus localized/regional degree of spread

#### 1. Males - Adjusting only for age

Results indicated an elevated odds ratio of 1.54 (1.14, 2.09) for regional and remote residential areas compared with a major city (Table 5).

**Table 5:** Age-adjusted odds ratios (95% CI) of males having distant/unknown liver cancer spread compared with localized/regional spread diagnosed during Sept 2016 – Dec 2018, according to sociodemographic predictors: linked NSW Cancer Registry and MADIP 2016 data

Characteristic	Localised /Regional (%)	Distant/ Unknown (%)	Age-adjusted odds ratios (95% CI)	Multivariate-adjusted odds ratios (95% CI)
**Total**	529 (100.0)	341 (100.0)		
**Residential remoteness**:				
Major cities	403 (76.2)	230 (67.4)	1.00	1.00
Regional/remote	126 (23.8)	111 (32.6)	1.54 (1.14, 2.09)	1.63 (1.20, 2.22)
**Country of birth**:				
Australia	286 (54.1)	200 (58.7)	1.00	
China	27 (5.1)	14 (4.1)	075 (0.38, 1.47)	
Italy, Greece, Lebanon	24 (4.5)	17 (5.0)	0.38 (0.04, 3.71)	
NZ, Philippines, Vietnam	36 (6.8)	19 (5.6)	0.75 (0.28, 2.05)	
United Kingdom/Other English speaking	54 (10.2)	31 (9.1)	0.80 (0.46, 1.39)	
Other Non-English speaking	102 (19.3)	60 (17.6)	0.82 (0.56, 1.18)	
**English speaking proficiency**:				
Poor	58 (11.0)	39 (11.4)	0.99 (0.64, 1.53)	
Not poor	471 (89.0)	302 (88.6)	1.00	
**Low-income household (<$26,000)**:				
Yes	162 (30.6)	115 (33.7)	1.12 (0.83, 1.49)	
No	367 (69.4)	226 (66.3)	1.00	
**Unemployed**:				
Yes	14 (2.6)	10 (2.9)	1.26 (0.55, 2.89)	
No	515 (97.4)	331 (97.1)	1.00	
**Labourer (occupation)**:				
Yes	37 (7.0)	16 (4.7)	0.74 (0.40, 1.37)	
No	492 (93.0)	325 (95.3)	1.00	
**Driver (occupation)**:				
Yes	39 (7.4)	22 (6.5)	1.01 (0.58, 1.76)	
No	490 (92.6)	319 (93.5)	1.00	
**Educational level (< year 12)**:				
Yes	198 (37.4)	132 (38.7)	1.08 (0.82, 1.43)	
No	331 (62.6)	209 (61.3)	1.00	
**No education**:				
Yes	13 (2.5)	11 (3.2)	1.28 (0.56, 2.89)	
No	516 (97.5)	330 (96.8)	1.00	
**Renting from housing authority**:				
Yes	49 (9.3)	31 (9.1)	1.03 (0.64, 1.66)	
No	480 (90.7)	310 (90.9)	1.00	
**Sole person household**:				
**Yes**	101 (19.1)	80 (23.5)	1.29 (0.92, 1.80)	
**No**	428 (80.9)	261 (76.5)	1.00	
**Disability (6 + months/ages < 70 yrs.)**:				
Yes	51 (9.6)	44 (12.9)	1.54 (0.99, 2.38)	1.72 (1.10, 2.70)
No	478 (90.4)	297 (87.1)	1.00	1.00
**Carer of person with disability**:				
Yes	51 (9.6)	35 (10.3)	1.08 (0.68, 1.70)	
No	478 (90.4)	306 (89.7)	1.00	
**SA2 area disadvantage**:				
Most disadvantage (Q1)	144 (27.2)	104 (30.5)	1.40 (0.91, 2.17)	
Quintile (Q2)	115 (21.7)	90 (26.4)	1.52 (0.97, 2.38)	
Quintile (Q3)	110 (20.8)	64 (18.8)	1.13 (0.71, 1.81)	
Quintile (Q4)	72 (13.6)	35 (10.3)	0.90 (0.53, 1.5)	
Quintile (Q5)	88 (16.6)	48 (14.1)	1.00	
**No. of conditions medicated**:				
0	41 (7.8)	26 (7.6)	1.56 (0.83, 2.92)	
1	49 (9.3)	28 (8.2)	1.43 (0.78, 2.62)	
2	89 (16.8)	37 (10.9)	1.00	
3	62 (11.7)	56 (16.4)	2.16 (1.27, 3.66)	
4	75 (14.2)	49 (14.4)	1.48 (0.87, 2.52)	
5	61 (11.5)	43 (12.6)	1.56 (0.90, 2.71)	
6–7	91 (17.2)	65 (19.1)	1.51 (0.91, 2.51)	
8+	61 (11.5)	37 (10.9)	1.25 (0.70, 2.22)	

##### Multivariable age-adjustment

Results were similar with substantive elevated odds of 1.63 (1.20, 2.22) for regional and remote areas compared with a major city. Also, an elevation of 1.72 (1.10, 2.70) applied when having a disability of 6 + months duration at age < 70 years.

#### 2. Females - Adjusting only for age

Results did not point to variations in odds of distant/unknown stage for the independent variables when adjusting only for age (Table 6).

**Table 6:** Age-adjusted odds ratios (95% CI) of females having distant/unknown liver cancer spread compared with localized/regional spread diagnosed during Sept 2016 – Dec 2018, according to sociodemographic predictors: linked NSW Cancer Registry and MADIP 2016 data

Characteristic	Localised /Regional (%)	Distant/ Unknown (%)	Age-adjusted odds ratios (95% CI)	Multivariate-adjusted odds ratios (95% CI)
**Total**	174 (100.0)	141 (100.0)		
**Residential remoteness**:				
Major cities	134 (77.0)	104 (73.8)	1.00	
Regional/remote	40 (23.0)	37 (26.2)	1.13 (0.65, 1.99)	
**English speaking proficiency**:				
Poor	34 (19.5)	19 (13.5)	0.59 (0.32, 1.09)	0.39 (0.18, 0.80)
Not poor	140 (80.5)	122 (86.5)	1.00	1.00
**Low-income household (<$26,000)**:				
Yes	52 (29.9)	42 (29.8)	0.98 (0.60, 1.60)	
No	122 (70.1)	99 (70.2)	1.00	
**Educational level (< year 12)**:				
Yes	84 (48.3)	73 (51.8)	1.06 (0.67, 1.67)	
No	90 (51.7)	68 (48.2)	1.00	
**Renting from housing authority**:				
Yes	16 (9.2)	12 (8.5)	0.96 (0.44, 2.12)	
No	158 (90.8)	129 (91.5)	1.00	
**Sole person household**:				
**Yes**	43 (24.7)	37 (26.2)	1.01 (0.60, 1.69)	
**No**	131 (75.3)	104 (73.8)	1.00	
**Disability (6 + months/ages < 70 yrs.)**:				
Yes	11 (6.3)	12 (8.5)	1.77 (0.73, 4.24)	
No	163 (93.7)	129 (91.5)	1.00	
**Carer of person with disability**:				
Yes	24 (13.8)	19 (13.5)	1.09 (0.56, 2.10)	
No	150 (86.2)	122 (86.5)	1.00	
**SA2 area disadvantage**:				
Most disadvantage (Q1)	48 (27.6)	53 (37.6)	0.96 (0.50, 1.85)	
Quintile (Q2)	35 (20.1)	31 (22.0)	0.76 (0.37, 1.55)	
Quintile (Q3)	38 (21.8)	13 (9.2)	0.30 (0.13, 0.68)	
Quintile (Q4)	25 (14.4)	13 (9.2)	0.45 (0.19, 1.05)	
Quintile (Q5)	28 (16.1)	31 (22.0)	1.00	
**No. of conditions medicated**:				
0 or 1 condition	19 (10.9)	23 (16.3)	2.09 (0.72, 6.11)	2.36 (0.84, 6.64)
2 conditions	27 (15.5)	14 (9.9)	1.00	0.93 (0.39, 2.21)
3	36 (20.7)	19 (13.5)	0.96 (0.40, 2.26)	1.00
4	27 (15.5)	13 (9.2)	0.83 (0.33, 2.12)	0.89 (0.37, 2.14)
5	19 (10.9)	17 (12.1)	1.49 (0.58, 3.80)	1.68 (0.71, 4.00)
6–7	29 (16.7)	30 (21.3)	1.67 (0.72, 3.88)	2.07 (0.96, 4.47)
8+	17 (9.8%)	25 (17.7)	2.27 (0.90, 5.70)	2.91 (1.25, 6.79)

##### Multivariable age-adjustment

This revealed elevated odds when 8 + other conditions were recorded of 2.91(1.25, 6.79) compared with the reference of 3 conditions. By comparison, poor English-speaking proficiency was associated with reduced odds of distant/unknown degree of spread at 0.39 (0.18, 0.80).

## Discussion

The present results complement those from previous studies with a broader range of sociodemographic and health characteristics that are associated with liver cancer and more advanced stage at diagnosis in NSW. These data will inform service planning and targeting. Repeating the process on a periodic basis, potentially in relation to future censuses, will indicate changes in risk profiles that may inform adjustments to service plans and priorities.

Previous studies have shown liver cancer rates in Australia to be associated with older age, male sex, lower area-based socioeconomic status, countries of birth outside Australia in Asia, and more recent time periods [1–3, 7]. The linked MADIP variables generally confirmed these earlier findings, supporting the likely validity of these data and the study design.

Results indicated higher age-adjusted odds of liver cancer by residential location in a major city, and by country of birth, with elevations for China, the Philippines and Vietnam, and less so, for Greece, Italy, and Lebanon, and New Zealand. Higher age-adjusted odds also applied to residents with poor English proficiency, low-income households, lower educational attainment, occupations of labourer or driver, those renting housing from housing authorities, sole person and sole female households, hose with poor English-speaking proficiency, low-income households, lower educational attainment levels than year 12, occupations of labourer or driver, those renting accommodation from housing authorities, sole person households, sole parent households (in females), jobless households with children, and those with a disability of six months or longer when aged under 70 years. These characteristics suggest that liver cancer generally occurs more frequently among residents experiencing other social and health disadvantage, thereby potentially compounding inequality. Irrespective of whether assessed at individual or SA2 residential area level, more socioeconomic disadvantage was associated with higher age-adjusted odds of liver cancer. The odds also were higher in those with a high number of medical conditions under medication.

Other results pointed to a higher proportion after age adjustment of distant or unknown degree of spread at diagnosis for residents of regional and more remote residential areas as opposed to a major city, those experiencing prolonged disability at age < 70 years, and in females, those with high numbers of other concurrent conditions. There were also some unexpected findings, including lower age-adjusted odds of liver cancer among those caring for a person with disability. These findings need further investigation and ideally, confirmation with data from other jurisdictions. They highlight the fact that correlates, while of potential value for service planning, may not have causal significance.

Data for Aboriginal and Torres Strait Islander residents were not presented in this paper due to small numbers. Larger numbers will be pursued in a further project in multi-jurisdictional analyses.

Preventive opportunities exist by targeting high-risk groups. These should be informed by the scientific literature. Examples would include, where relevant, the use of hepatitis B vaccination and promotion of healthy lifestyles to address risks from diabetes mellitus, being overweight and obese, having high alcohol consumption, being tobacco smokers, illicit drug users, and having unprotected sex [5, 6]. Carriers of hepatitis B and C infection should receive guidance on how best to avoid transmission of infection to uninfected partners and family members. Notably around half the burden of liver cancer in Australia have been attributed to hepatitis B and C infections, [5] such that treating and curing HCV and suppressing HBV infection with drugs would markedly reduce the risk if hepatocellular carcinoma [20–22].

The current study moves beyond behavioral risk profiles to document demographic and social characteristics that accompany liver disease. Those characteristics can be mapped throughout the community using census and other records and inform local area, preventive and early detection interventions. Local conversations on prevention can then focus on people and their circumstance rather than personal behavior at the outset.

Survival outcomes for liver cancer are poor, with a five-year relative survival approximating 22% in Australia [3, 4]. The potential to increase survival through earlier detection is indicated by evidence of smaller cancers diagnosed through surveillance that are more likely to have curative treatment, and where results indicate higher survival to persist after adjusting for lead time and related biases [22]. In addition, it would be desirable to seek care early, where possible from specialist clinical units experienced in the management of this disease. Five-year relative survival is higher with less extensive spread of this cancer at diagnosis, as indicated by NSW and international data, including USA SEER data indicating survival of 35% for localized stage, 12% for regional spread, and 3% where distant metastases apply [23].

Policy priorities for liver cancer include increasing awareness of risk factors, optimizing hepatitis B vaccine coverage for high-risk populations, including universal coverage for Aboriginal and Torres Strait Islander people, and for those migrating to Australia from the high-risk Asian and other countries identified in this report, and increasing access to early treatment and support for people infected with hepatitis B and C.

Positive features of this study were the ease with which de-identified linked routinely collected data could be used to better indicate the risk profiles of NSW population groups at increased risk of liver cancer, and those being diagnosed at a more advanced stage. NSW (and Australia) has long had well-developed population-based cancer and cancer management databases, [24] well defined data linkage protocols, and a network of data linkage units and remote-access laboratories to use these data safely [25, 26]. This framework needs further development to provide more comprehensive population-wide evidence to inform service planning, delivery and evaluation.

Limitations of this study include the combining of categories where numbers were low and point estimates were similar. This was a result-guided activity born of necessity to build numbers. Larger numbers should be pursued through multi-jurisdictional studies to gain more precise results without the need to combine categories. Another limitation was the lack of access in this study to linked population-wide data on prevalence of hepatitis B and C infection, hepatitis B vaccination, and risk factors such as overweight and obesity, diabetes, excess alcohol consumption, tobacco smoking, illicit drug use, unprotected sex, and medical conditions such as metabolic dysfunction associated fatty liver disease, cirrhosis and haemochromatosis [5, 6]. Linked data on diagnostic and clinical care pathways and supportive care were not available for use in this study to investigate disparities in service utilization and timeliness. Population-based data from biobanks and genomic databases also were not available, which could have increased the value of the linked data for exploring at population level the effects of biological factors at cellular and sub-cellular level. Future data collection and data linkage activity should address these limitations.

The present study included many comparisons, most of which were consistent in showing disparities in liver cancer incidence and staging patterns across population sub-groups, and many of which would have added to social hardship and health disadvantage. Confirmatory data from other states and territories would have strengthened these findings, especially where numbers were small and more open to random variation.

This study describes subgroups at increased of liver cancer and advanced diagnostic stages to inform service planning and benchmarking in NSW. It was not designed to investigate causation or produce novel epidemiological insights, although with broader data linkages there would be the potential to do so.

## Conclusions

This study demonstrated the ease with which linked routinely collected data could be used to identify sociodemographic disparities in liver cancer risk and more advanced stage in NSW. MADIP data holdings provide value for this purpose that add to that already available from the NSW Cancer Registry and Australia-wide through the Australian Institute of Health and Welfare and Australasian Association of Cancer Registries. The data assist insights into the social determinants and burdens of these cancers. In this study the data indicate that liver cancer places a heavier burden on those sectors of the NSW population already likely to be disproportionately affected by social hardships and health disadvantage. Less favorable stage-related prognostic profiles were observed in more remote populations and those already living with disability and numerous health conditions. These NSW-wide data are available to assist the planning of health and welfare services. NSW has advanced data linkage systems that can be used for this purpose. Capacity for data linkage is being extended across the Australian population to cover cancer management from primary prevention and screening through to cancer care and support along the cancer pathway. Further data development will support an evidence-based approach to cancer control, including assisting with priority setting, targeting of services, and establishing benchmarks.

### Electronic supplementary material

Below is the link to the electronic supplementary material.

Supplementary Material 1

## Data Availability

The original data for this study were provided by the Australia Bureau of Statistics, the Australian Department of Health and the NSW Ministry of Health following approval by relevant ethics committees. These data may be available to other researchers meeting the relevant data access and ethical requirements. Requests and enquiries on the data processing and analyses code for this article can be made to DB.
