# Supplementary material for: Demographic, health, and prognostic characteristics of Australians with liver cancer: a cohort study of linked data in New South Wales for informing cancer control
Source: BMC Public Health. 2023 Oct 9;23:1957. doi: 10.1186/s12889-023-16809-y (PMC10563226; doi:10.1186/s12889-023-16809-y)
Supplement: Supplementary file 1 — Supplementary Material 1 [file 12889_2023_16809_MOESM1_ESM.docx]

**Appendix A: Age-adjusted odds ratios (95% CI) of liver cancer diagnosed during Sept 2016 – Dec 2018, according to sociodemographic predictors: linked NSW Cancer Registry and MADIP data**

| **Supplement to**  **Table 2 males** | **Numbers in liver cancer cohort** | **Numbers of community controls** | **Age-adjusted odds ratios (95% CI)** |
| --- | --- | --- | --- |
| **Affective condition medications**  Yes  No | 194  676 | 288 441  2 192 428 | 1.59 (1.35, 1.87)  1.00 |
| **Antipsychotic medications:**  Yes  No | 36  834 | 50 438  2 430 431 | 1.68 (1.20, 2.35)  1.00 |
| **GP consultations (in 12m):**  0  1-3  4-7  8-16  17-35  36+ | 19  91  193  338  193  36 | 496 616  846 473  628 915  399 422  98 770  10 673 | 1.00  2.48 (1.51, 4.07)  5.69 (3.55, 9.14)  12.44 (7.79, 19.86)  23.41 (14.46, 37.87)  39.25 (22.30, 69.07) |
| **Mental health plan:**  None  1 event  2+ events | 798  53  19 | 2 339 619  101 322  39 928 | 1.00  2.16 (1.63, 2.86)  1.91 (1.21, 3.02) |
| **Health assessments:**  None  1+ | 788  82 | 2 387 411  93 458 | 1.00  0.85 (0.67, 1.08) |
| **Chronic disease plan:**  None  1 event  2 events  3+ events | 477  45  173  175 | 2 100 810  66 210  201 656  112 193 | 1.00  1.60 (1.17, 2.18)  2.05 (1.71, 2.45)  3.19 (2.65, 3.83) |

| **Supplement to**  **Table 3 females** | **Numbers in liver cancer cohort** | **Numbers of community controls** | **Age-adjusted odds ratios (95% CI)** |
| --- | --- | --- | --- |
| **Affective condition medications**  Yes  No | 98  217 | 570 283  2 370 981 | 1.30 (1.02, 1.65)  1.00 |
| **Antipsychotic medications:**  Yes  No | 18  297 | 60 187  2 881 077 | 1.75 (1.08, 2.82)  1.00 |
| **GP consultations (in 12m):**  0  1-3  4-7  8-16  17-35  36+ | 11  15  74  140  64  11 | 313 337  813 263  912 355  714 867  172 376  15 066 | 1.00  0.63 (0.29, 1.39)  2.35 (1.25, 4.44)  4.30 (2.33, 7.95)  6.03 (3.18, 11.43)  10.39 (4.50, 24.00) |
| **Mental health plan:**  None  1+ event | 295  37 | 2 649 916  291 348 | 1.00  0.84 (0.47, 1.50) |
| **Health assessments:**  None  1+ | 278  37 | 2 809 935  131 329 | 1.00  0.78 (0.54, 1.11) |
| **Chronic disease plan:**  None  1 event  2 events  3+ events | 155  17  80  63 | 2 394 525  84 656  300 665  161 418 | 1.00  1.38 (0.83, 2.30)  2.07 (1.57, 2.73)  2.56 (1.89, 3.46) |
